# Parathyroid carcinoma in a 13-year-old girl with a long-term survival

**DOI:** 10.1186/s40792-020-00914-w

**Published:** 2020-06-22

**Authors:** Yoko Omi, Tomoko Yamamoto, Yoji Nagashima, Koichiro Abe, Kumiko Karasawa, Yukichi Tanaka, Takahiro Okamoto

**Affiliations:** 1 Department of Breast and Endocrine Surgery, Tokyo Women’s Medical University, 8-1 Kawada-cho, Shinjuku-ku, Tokyo, Japan; 2 Department of Diagnostic Pathology, Tokyo Women’s Medical University, 8-1 Kawada-cho, Shinjuku-ku, Tokyo, Japan; 3 Department of Diagnostic Imaging and Nuclear Medicine, Tokyo Women’s Medical University, 8-1 Kawada-cho, Shinjuku-ku, Tokyo, Japan; 4 Department of Radiation Oncology, Tokyo Women’s Medical University, 8-1 Kawada-cho, Shinjuku-ku, Tokyo, Japan; 5 Department of Diagnostic Pathology, Kanagawa Children’s Medical Center, 2-138-4 Mutsukawa, Minami-ku, Yokohama, Japan

**Keywords:** Parathyroid carcinoma, Child, Positron emission tomography of methionine, Denosumab, Radiation

## Abstract

**Background:**

Parathyroid carcinoma as a cause of primary hyperparathyroidism in children is extremely rare. We report a case of parathyroid carcinoma which occurred in a 13-year-old girl who survived for more than 45 years after the first operation.

**Case presentation:**

A woman was admitted to our hospital for the treatment of recurrent parathyroid carcinoma in the neck. She had been diagnosed with primary hyperparathyroidism from a fibula fracture and underwent parathyroidectomy at 13 years old. She had no family history of multiple endocrine neoplasia or jaw tumor syndrome. Genetic testing was not performed, and the histopathological diagnosis of the tumor had been parathyroid adenoma at the time. At 22 years old, she showed hypercalcemia after a femur fracture. Pulmonary metastases of parathyroid carcinoma in the bilateral lungs were found and surgically removed. Regarding the clinical course, her diagnosis was corrected from parathyroid adenoma to parathyroid carcinoma. At 33 years old, re-resection of the lung metastases was performed. For 10 years, her serum calcium level stayed within the normal range. However, her serum calcium level and intact parathyroid hormone eventually began to increase. Two masses suspected of being parathyroid carcinoma recurrence were found in the neck when she was 57 years old. En bloc resection was performed. Pathologically, the tumors were diagnosed as parathyroid adenoma. The serum calcium level and intact parathyroid hormone did not decrease after the operation. A ^99m^Tc-methoxy-isobutyl-isonitrile- and ^18^F-fluorodeoxyglucose-negative, ^11^C-methionine-positive tumor was detected at the right side of the trachea in the neck. The tumor was removed, along with the thyroid, muscle, and trachea that were involved. The pathological diagnosis was parathyroid carcinoma recurrence. The serum calcium level and intact parathyroid hormone decreased temporarily but had increased again 8 months later. Methionine-positive tumors were found at the right side of the trachea and suspected of being a recurrence. Denosumab reduced her serum calcium level, and radiation successfully suppressed the growth of the recurrent tumors.

**Conclusion:**

We have reported a rare case of parathyroid carcinoma in a child who has survived for over 40 years. Positron emission tomography of ^11^C-methionine was useful for detecting local recurrence. This patient’s long-term survival has been attributed to multimodality treatment including repeated surgery, medication, and radiation.

## Background

Parathyroid carcinoma (PC) as a cause of primary hyperparathyroidism (PHPT) in children is extremely rare. We herein report a case of recurrent PC in a 57-year-old woman who survived for more than 45 years after the first operation.

## Case presentation

**Fig. 1:**
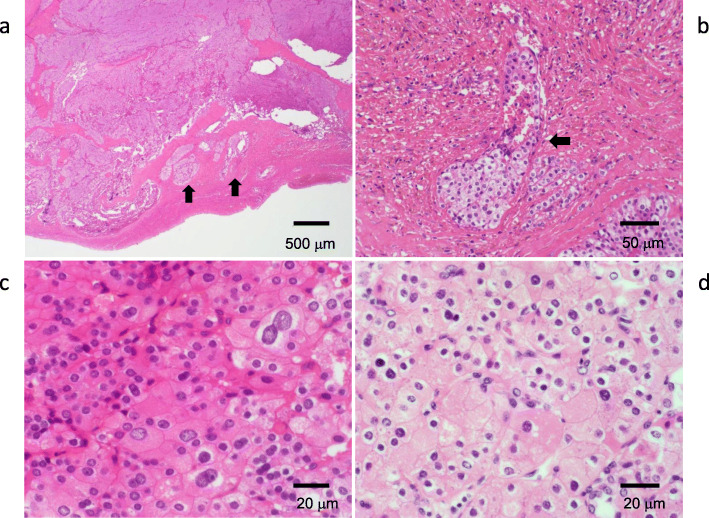
Histological findings of the parathyroid tumor removed when the patient was 13 years old and the lung metastasis removed when she was 33 years old (hematoxylin and eosin stain). **a** Capsular invasion of the tumor (arrows). **b** Vascular invasion of the tumor cells (arrow). **c** Pleomorphic tumor cells with a large nucleus. **d** Tumor cells with pleomorphic nuclei showed solid growth, just like the primary tumor

A 57-year-old woman was admitted to our hospital for the treatment of recurrent PC. She had been diagnosed with primary hyperparathyroidism from a fibula fracture and had her right lower parathyroid gland (15 g in weight) removed at the Kanagawa Children’s Medical Center when she was 13 years old. The pathological diagnosis at the time had been parathyroid adenoma [1]. She had no family history of multiple endocrine neoplasia or jaw tumor syndrome. Genetic testing was not performed. The parathyroid specimen of the first operation had been microscopically reviewed for this case report, after 44 years from the operation. The tumor cells have been found to be pleomorphic with infiltration into the capsule and vessels (Fig. 1a–c). Based on these findings, the specimen was determined to be PC.

At 22 years old, she showed hypercalcemia (serum calcium level, 18 mg/dL) after a femur fracture at another hospital. Recurrent disease was not found in the neck, but lung metastases were discovered. Regarding the clinical course, her diagnosis was corrected from parathyroid adenoma to PC at this time. The pulmonary metastases in the bilateral lungs were removed surgically.

**Fig. 2:**
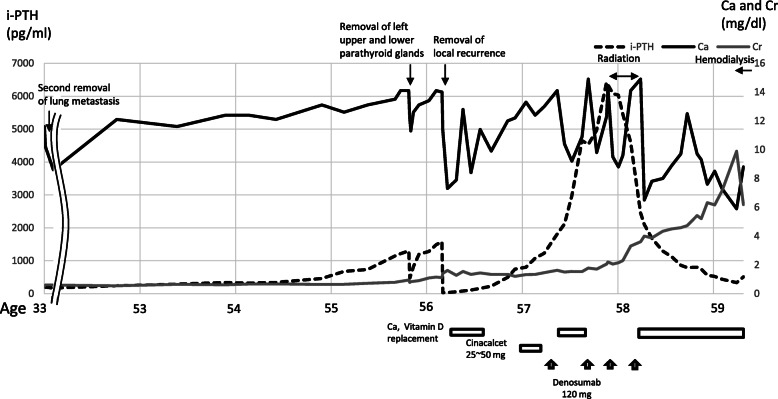
Blood test results and progress of treatment

**Fig. 3:**
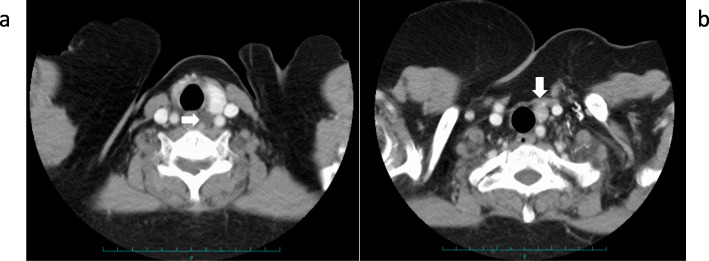
CT findings of the neck. **a** A mass found behind the left thyroid lobe (arrow). **b** A mass found caudally next to the lower pole of the left thyroid lobe (arrow)

**Fig. 4:**
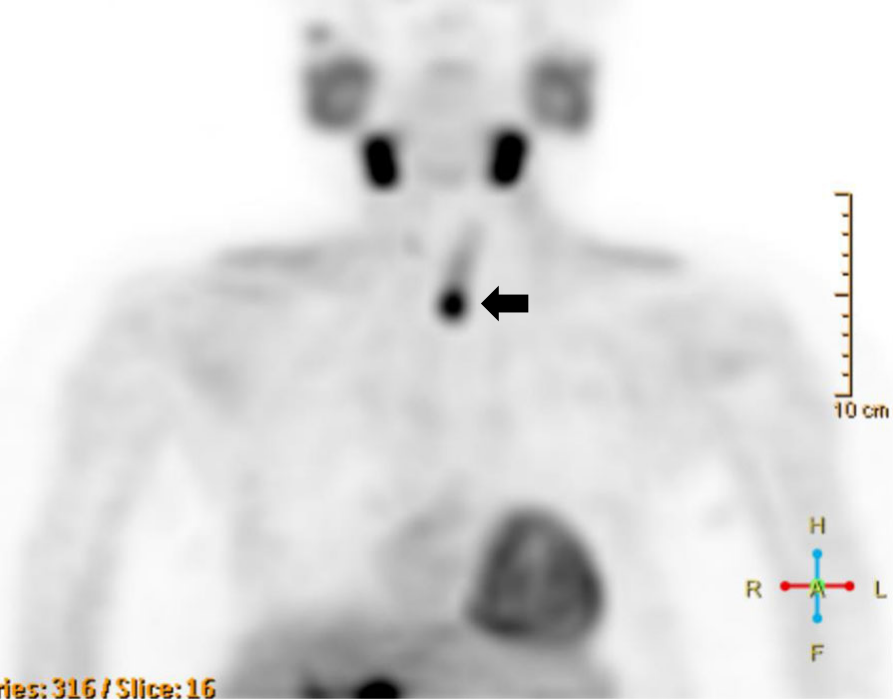
MIBI scintigraphy findings. MIBI accumulated in the lower mass (arrow) but not in the upper mass

**Fig. 5:**
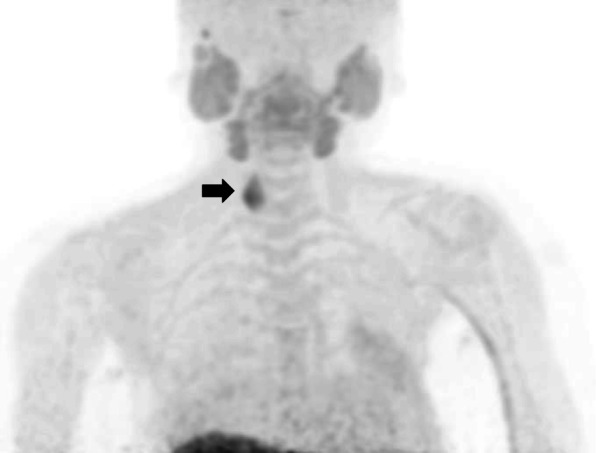
^11^C-methionine PET-CT findings after the removal of the upper and lower parathyroid glands. Methionine-positive tumor right next to the trachea (arrow)

At 33 years old, re-resection of the lung metastases was performed in our hospital. The histopathology of the lung metastases resembled that of the first operation (Fig. 1d). This case was reported in the case series of Fujimoto et al. [1] when the patient was 35 years old. For 10 years, her serum calcium level remained within the normal range. However, her serum calcium level and intact parathyroid hormone (i-PTH) gradually began to increase, reaching 14.2 (reference range, 8.5 to 9.9) mg/dL and 1302 (reference range, 16 to 65) pg/mL when she was 57 years old (Fig. 2). Two masses were found behind the left thyroid lobe (Fig. 3a) and caudally next to the left thyroid lobe (Fig. 3b) by computed tomography (CT) and suspected of being a PC recurrence. ^99m^Tc-methoxy-isobutyl-isonitrile (MIBI) accumulated in the lower mass but not in the upper mass (Fig. 4). En bloc resection of those masses was performed. Pathologically, both tumors were diagnosed as parathyroid adenoma, as they did not show any malignant features. However, the operation failed to reduce her serum calcium level and i-PTH sufficiently, with persistent levels of 12.2 mg/dL and 475 pg/mL, respectively (Fig. 2). Positron emission tomography (PET) using ^18^F-fluorodeoxyglucose (FDG) was performed, but it failed to reveal residual disease. An additional examination by ^11^C-methionine PET was therefore performed, and a methionine-positive tumor was found at the right side of the trachea in the neck (Fig. 5).

**Fig. 6:**
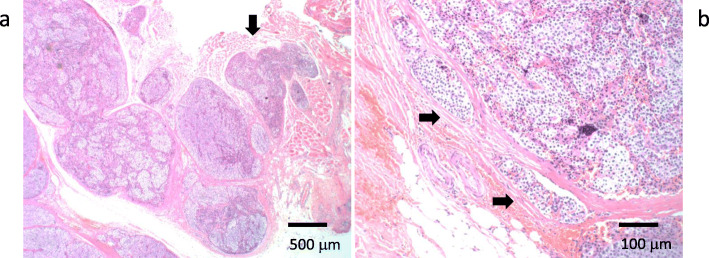
Histological findings of the recurrent tumor right next to the trachea (hematoxylin and eosin stain). **a** The tumor invaded through the muscle (arrow). **b** The tumor invaded the vessels (arrows)

**Fig. 7:**
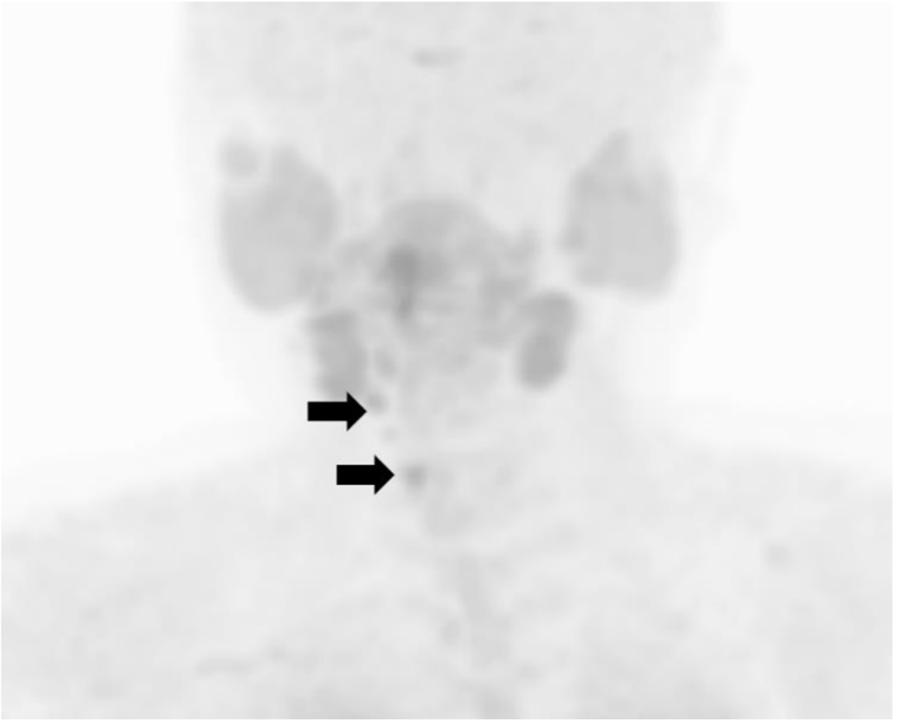
^11^C-methionine PET-CT findings after the removal of local recurrence. Methionine-positive tumors right next to the trachea (arrows)

As tumor invasion into the thyroid, cricothyroid muscle, and membrane between the tracheal cartilages was recognized during the operation, it was removed, along with the involved surrounding tissues. After this surgery, both the serum calcium and i-PTH levels declined to 7.7 mg/dL and 28 pg/mL, respectively (Fig. 2). She was administered calcium lactate and calcitriol to maintain her serum calcium concentration. She had never experienced symptoms due to recurrent laryngeal nerve paralysis. A histopathologic examination confirmed the diagnosis of PC recurrence (Fig. 6a, b). On day 7 after the operation, she developed a fever and arthralgia in the ankles and knees and was diagnosed with pseudogout. Her hemoglobin declined to 6.8 g/dL on day 14 after the operation. Chronic inflammation due to prolonged pseudogout and renal dysfunction due to hypercalcemia were thought to be the cause. Her anemia resolved spontaneously after her pseudogout was cured. Her serum calcium and i-PTH levels gradually increased again. Replacement of calcium was discontinued at 5 months after the operation. When the serum calcium level reached 12 mg/dL and i-PTH became 496 pg/ml at 8 months after the surgery (Fig. 2), CT and MIBI scintigraphy were performed again, but no recurrence was detected. However, ^11^C-methionine PET revealed two masses at the right side of the trachea (Fig. 7). Recurrence of PC was suspected. She refused surgery this time. Cinacalcet (25 mg) was administered for 4 months but stopped because of gastrointestinal symptoms. Denosumab was started, and it successfully reduced her serum calcium level to 7.7 mg/dL (Fig. 2).

**Fig. 8:**
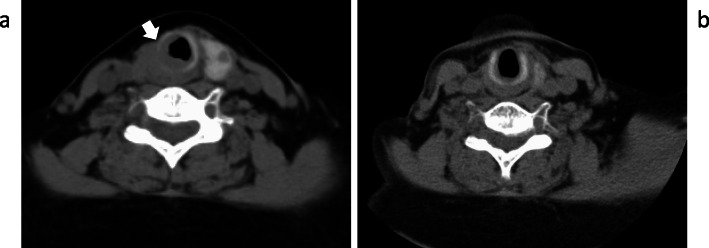
CT findings before and after radiation. **a** Stenosis of the trachea occurred due to recurrent tumor invasion (arrow). **b** The recurrent tumor could not be recognized 1 year after radiation therapy.

While the injection of denosumab every 3 months kept her calcium levels low or in the normal range, methionine PET showed that the recurrent tumor at the right side of the trachea had grown compared to the proportions detected 10 months previously. Airway stenosis due to the tumor invasion into the trachea was observed by CT (Fig. 8a). The i-PTH level increased to 6075 pg/mL. Radiation (60 Gray/30 fractions) for local recurrence was performed. The i-PTH level started to decrease at the end of the treatment (Fig. 2). The tumor began to shrink after 1 month of treatment, and both the tumor size and i-PTH level remained low at 1 year after radiation therapy (Figs. 2 and 8b). The serum calcium level is being controlled without denosumab following radiation therapy, although hemodialysis was initiated due to renal failure, which developed as a result of long-term hypercalcemia (Fig. 2).

## Discussion

We identified 17 cases of PC in children under 16 years old in the literature [1–17] (Table 1). Very high levels of serum calcium and i-PTH, a palpable neck mass, and severe clinical symptoms are the specific clinical features of PC [18] and seem to be characteristic of PC in children as well. Compared to PHPT in adults, PHPT in children is reportedly more symptomatic [19], with a greater incidence of hypercalcemia and hypercalciuria at similar concentrations of i-PTH [20]. PC appearing in children often suggests the involvement of hereditary diseases, such as hyperparathyroidism-jaw tumor syndrome. Indeed, three previous pediatric patients had a family history of PHPT [4, 6, 7], and two had a confirmed germline *CDC73* mutation [12, 15].

**Table 1:** Clinical literature review of parathyroid carcinoma in children under 16

Year	Sex	Age	Ca, mg/dL	i-PTH, pg/mL	Symptoms	Family history	Palpable neck mass	Operation	Outcome
1973 [2]	–	13	–	–	–	–	–	–	–
1984 [3]	M	15	112.6	–	Pancreatitis	–	No	PTx	–
1984 [1] This case	F	13	15.9	–	Bone pain	No	–	PTx	Lung meta at 9, 20 yrs Local rec at 42 yrs
1993 [4]	M	14	13.2	353	Bone pain	Yes	Yes	En bloc resection	No rec after 2 yrs
1999 [5]	M	15	20.7	154	Fatigue, nausea, weight loss	–	Yes	En bloc resection	No rec after 1 yr
2002 [6]	F	8	14.3	82	Renal stone	Yes	Yes	PTx	No rec after 16 ms
2009 [7]	M	10	15.5	–	Anorexia, fatigue, knee pain	Yes	No	Mediastinal PTx, thymectomy	No rec after 18 ms
2011 [8]	F	14	14.3	2792	Arthralgia	No	–	HTx	No rec after 18 ms
2012 [9]	F	13	12.0	8363	None	No	Yes	En bloc resection	Lung meta after 6 ms
2012 [10]	M	11	–	1630	Bowing legs with pain	–	–	PTx→HTx	–
2015 [11]	F	10	12.2	2217	Abdominal pain, joint deformity	–	–	–	–
2016 [12]	F	8	12.5	453	–	No	–	PTx	Local rec at 5 yrs Lung meta at 5 yrs
2016 [13]	M	14	17.1	1164	Hip pain	No	Yes	En bloc resection	No rec after 1 yr
2016 [14]	F	14	13.4	1013	Leg pain	No	Yes	En bloc resection	No rec after 6 ms
2019 [15]	F	15	15.8	1170	Fever, fatigue	No	Yes	En bloc resection	No rec after 2 yrs
2020 [16]	M	13	15.4	980	Polyuria, polydipsia, leg pain	No	–	En bloc resection	No rec after 1.5 yrs
2020 [17]	M	10	14.3	1075	Back pain	–	–	PTx	–

*Ca* calcium, *i-PTH* intact parathyroid hormone, *BMD* bone mineral density, *HPT* hyperparathyroidism, *yr* year, *m* month, *PTx* parathyroidectomy, *HTx* hemithyroidectomy, *meta* metastasis, *rec* recurrence

Ultrasonography, MIBI scintigraphy, or CT is usually used to detect the localization of a hyperfunctioning parathyroid. In cases of malignancy, FDG-PET can be used to stage or detect recurrence in PC patients [21]. In the present case, however, FDG did not accumulate in the lesion of local recurrence right next to the trachea. For MIBI-negative PHPT cases, ^11^C-methionine PET is reported to be useful [22]. Indeed, methionine PET successfully detected MIBI- and FDG-negative PC recurrence in the present patient.

According to Schantz and Castleman [2], the histopathological diagnosis of PC is made based on the distinctive histological features, such as the presence of a trabecular pattern, mitosis, thick fibrous bands, and capsular or vascular invasion. The pathological findings are not always described in detail in the reported cases, but these criteria can be used to diagnose PC in children, as there are no characteristic pathological features of PC in children.

Among the 17 cases previously reported [1–17], 3 patients [1, 9, 15] (17.6%) experienced recurrence. In our series of 38 patients [23], including the present case, 9 patients (23.7%) experienced recurrence within 5 years, and 15 (39%) developed persistent or recurrent PC after the initial surgery (within 0–144 months; median, 31 months). The recurrence rate of PC does not seem to differ markedly between children and adults. The 5-year survival rate for PC in our series was 90% [23]. As none of the reported cases was followed for a long time, none of the children died from PC. The present case is the only one that has been followed for more than 5 years. To ensure a good prognosis, local control via complete resection of the primary tumor is important [2]. If the cancer relapses, recurrence including distant metastasis should be removed operatively [24]. Indeed, resection of pulmonary metastasis was performed in all cases with recurrence [1, 9, 15]. There is no effective therapy at present for unresectable PC [25]. In some cases, anti-PTH immunotherapy has been able to successfully manage PC [26, 27]. Advances in medical treatment for PC, including anti-PTH immunotherapy, are expected [25].

Management of hypercalcemia due to recurrent PC is also important. Cinacalcet has proven effective for the treatment of hypercalcemia in recurrent PC [28]. Cinacalcet was used in a child with pulmonary metastasis [12], but there is not enough evidence of efficacy and safety for using cinacalcet for children. Bisphosphonate and denosumab are also useful for reducing calcium levels. Denosumab was selected for the present patient as she suffered from renal dysfunction. There have been some reports of denosumab successfully controlling hypercalcemia due to recurrent PC that was resistant to cinacalcet or bisphosphonate [17, 29–31]. Indeed, our patient’s serum calcium level was effectively decreased by denosumab.

PC is usually radioresistant. A few small case reports have described the application of radiation therapy in an adjuvant setting, but evidence supporting the efficacy of radiation for recurrent PC is even more limited than that for adjuvant therapy [32]. Rasmuson et al. reported a PC case with pulmonary metastasis that irradiation successfully decreased tumor size and serum calcium and i-PTH levels [33]. This case also suggests the efficacy of palliative high-dose radiation therapy for uncontrolled hypercalcemia due to recurrent PC. Long-term survival was achieved thanks to multimodality treatment combining repeated surgery for metastasis and recurrence, medical therapy for serum calcium control, and radiation therapy.

## Conclusion

PC is extremely rare in children. We reported a case of PC that initially occurred in a 13-year-old girl who has since survived for more than 40 years with her disease. ^11^C-methionine PET was useful for detecting local recurrence. This patient’s long-term survival has been attributed to multimodality treatment.

## Data Availability

All data generated or analyzed during this study are included in this published article.

## Abbreviations

CT: Computed tomography
FDG: ^18^F-fluorodeoxyglucose
i-PTH: Intact parathyroid hormone
MIBI: ^99m^Tc-methoxy-isobutyl-isonitrile
PC: Parathyroid carcinoma
PET: Positron emission tomography
PHPT: Primary hyperparathyroidism
